# The Oncolytic Avian Reovirus p17 Protein Inhibits Invadopodia Formation in Murine Melanoma Cancer Cells by Suppressing the FAK/Src Pathway and the Formation of theTKs5/NCK1 Complex

**DOI:** 10.3390/v16071153

**Published:** 2024-07-17

**Authors:** Chao-Yu Hsu, Jyun-Yi Li, En-Ying Yang, Tsai-Ling Liao, Hsiao-Wei Wen, Pei-Chien Tsai, Tz-Chuen Ju, Lon-Fye Lye, Brent L. Nielsen, Hung-Jen Liu

**Affiliations:** 1 Division of Urology, Department of Surgery, Tungs’ Taichung MetroHarbor Hospital, Taichung 435, Taiwan; t4361@ms.sltung.com.tw; 2 Ph.D. Program in Translational Medicine, National Chung Hsing University, Taichung 402, Taiwan; tlliao1972@gmail.com (T.-L.L.); ptsai@dragon.nchu.edu.tw (P.-C.T.); 3 Institute of Molecular Biology, National Chung Hsing University, Taichung 402, Taiwantcju@dragon.nchu.edu.tw (T.-C.J.); 4 Department of Medical Research, Taichung Veterans General Hospital, Taichung 407, Taiwan; 5 Department of Food Science and Biotechnology, National Chung Hsing University, Taichung 402, Taiwan; hwwen@nchu.edu.tw; 6 Department of Life Sciences, National Chung Hsing University, Taichung 402, Taiwan; 7 Department of Medical Research, Tungs’ Taichung MetroHarbor Hospital, Taichung 435, Taiwan; lonfyelye@gmail.com; 8 Department of Microbiology and Molecular Biology, Brigham Young University, Provo, UT 84602, USA; bnielsen28@gmail.com; 9 The iEGG and Animal Biotechnology Center, National Chung Hsing University, Taichung 402, Taiwan; 10 Rong Hsing Research Center for Translational Medicine, National Chung Hsing University, Taichung 402, Taiwan

**Keywords:** avian reoviruses, p17, p53/PTEN, FAK/Src, TKs5/NCK1 complex, Rab40b, MMP9, invadopodia

## Abstract

To explore whether the p17 protein of oncolytic avian reovirus (ARV) mediates cell migration and invadopodia formation, we applied several molecular biological approaches for studying the involved cellular factors and signal pathways. We found that ARV p17 activates the p53/phosphatase and tensin homolog (PTEN) pathway to suppress the focal adhesion kinase (FAK)/Src signaling and downstream signal molecules, thus inhibiting cell migration and the formation of invadopodia in murine melanoma cancer cell line (B16-F10). Importantly, p17-induced formation of invadopodia could be reversed in cells transfected with the mutant PTEN_C124A_. p17 protein was found to significantly reduce the expression levels of tyrosine kinase substrate 5 (TKs5), Rab40b, non-catalytic region of tyrosine kinase adaptor protein 1 (NCK1), and matrix metalloproteinases (MMP9), suggesting that TKs5 and Rab40b were transcriptionally downregulated by p17. Furthermore, we found that p17 suppresses the formation of the TKs5/NCK1 complex. Coexpression of TKs5 and Rab40b in B16-F10 cancer cells reversed p17-modulated suppression of the formation of invadopodia. This work provides new insights into p17-modulated suppression of invadopodia formation by activating the p53/PTEN pathway, suppressing the FAK/Src pathway, and inhibiting the formation of the TKs5/NCK1 complex.

## 1. Introduction

Invadopodia are actin-rich protrusions of the plasma membrane that are associated with sites of proteolytic degradation of the extracellular matrix (ECM) in cancer invasiveness and metastasis [1,2]. Invadopodia are found in invasive cancer cells and are important for their ability to invade through the ECM [3]. Invadopodia are generally visualized by the holes they create in ECM-coated plates, in combination with immunohistochemistry for the invadopodia localizing proteins including actin, cortactin, and tyrosine kinase substrate 5 (Tks5) [1,2,4]. The large scaffolding protein TKs5 is phosphorylated by Src kinase and is important for invadopodia maturation and formation [5,6]. Tks5 functions as a tether mediating the targeting of transport vesicles containing matrix metalloproteinases (MMPs; MMP2 and MMP9) and Tks5 to the extending invadopodia [7]. A previous study suggested that the small GTPase Rab40b levels are increased in metastatic breast cancers and are required for MMP2 and MMP9 secretion from the invadopodia in breast cancer cells [8]. It is also crucial for breast tumor growth and metastasis in vivo [8]. Furthermore, a recent study revealed that the Rab40b-TKs5-dependent transport pathway mediates invadopodia extension [7]. MMPs are able to degrade many components of ECM and are important for normal processes, including tissue remodeling and wound healing. The matrix degradation activity of invadopodia is attributed to the targeted secretion of matrix-degrading enzymes such as MMPs. MMP2, MMP9, and MMP14 have been shown to enhance cancer progression due to their ability to degrade basement membrane components. These MMPs are enriched at the invadopodia and are necessary for cancer metastasis [9,10,11,12,13,14,15]. It was reported that MMP2 and MMP9 are not transported to invadopodia by endosomes, but instead are targeted directly from the Golgi [8] through microtubule motors (kinesin) and by actin regulators (cortactin), suggesting that these MMPs are targeted to invadopodia through at least two different membrane transport pathways.

Avian reovirus (ARV) is nonenveloped and belongs to the family Reoviridae. Several reports have demonstrated that ARV is an oncolytic virus which induces apoptosis of cancer cells, regulates cell immune response, and exposes tumor-associated antigens to the immune system [16,17,18,19,20,21,22]. ARV has 10 double-stranded RNA genome segments. It can replicate in the cytoplasm of infected cells. The S1 genome segment of ARV has three open reading frames that are translated into proteins σC, p10, and p17, respectively. It was found that p17 is a nucleocytoplasmic shuttling protein [23] that has been demonstrated to regulate several cellular signal pathways to modulate cell cycle retardation, the formation of autophagy, angiogenesis, viral protein synthesis, and virus replication [23,24,25,26,27,28]. To date, although recent studies have suggested that p17 retards the cell cycle of several cancer cell lines [17,25], reduces tumor size in vivo [17], and suppresses angiogenesis by promoting DPP4 secretion [21], it is unclear whether p17 protein modulates cell migration and the formation of invadopodia. The aim of this work was to perform a comprehensive study to investigate whether p17 protein modulates cell migration and the formation of invadopodia and its involved mechanisms. This work reveals, for the first time, that p17 suppresses the formation of invadopodia in B16-F10 cancer cells by activation of the p53/PTEN pathway, inhibition of the FAK/Src pathway, and enhancement of the formation of the TKs5/ non-catalytic region of tyrosine kinase adaptor protein 1 (NCK1) complex.

## 2. Materials and Methods

### 2.1. Virus and Cells

In the present study, the S1133 strain of ARV was used. Murine melanoma cancer cell line (B16-F10) [19] was cultured in Dulbecco’s modified Eagle’s Medium (DMEM) with 5–10% heat-inactivated fetal bovine serum (Hyclone, Logan, UT, USA) and 1% penicillin (100 IU/mL)/streptomycin (100 g/mL) (Gibco, Grand Island, NY, USA). B16-F10 cells were propagated in a 37 °C, 5% CO_2_ humidified incubator. Cells were seeded in 10 cm culture dishes one day before each experiment until they reached about 75% confluence.

### 2.2. Reagents and Antibodies

The Akt III inhibitor, specific for Akt, was purchased from Enzo Life Science (New York, NY, USA). To examine whether p17-modulated decreased levels of TKS5 are regulated by the ubiquitin-proteasome-mediated degradation pathway, cells were transfected with the pCI-neo-p17 plasmid DNA for 6 h followed by treatment with MG132 (1 mM). MG132 was from Calbiochem Co. (San Diego, CA, USA). Gelatin and Alexa-Fluor-gelatin were from Cell Signaling (Danvers, MA, USA). The catalog numbers and dilution factors of the primary and secondary antibodies used in this study are shown in Supplementary Table S1. Antibodies against ARV p17 protein were our laboratory stocks [17].

### 2.3. Reverse Transcription (RT), Polymerase Chain Reaction (PCR) and Plasmid Construction

Total RNA was extracted from B16-F10 cells using TRIzol kit (Thermo Fisher Scientific Inc. Waltham, MA, USA) based on the manufacturer’s procedures. The primer pairs used in this work are shown in Supplementary Table S2. The PCR products were subcloned into the corresponding sites of the pcDNA3.1 vector. In the present study, RT was performed at 42 °C for 15 min and 72 °C for 15 min. PCR was performed with 1 μL of cDNA, 1 μL of each primer, 2 μL of PCR mix, and 15 μL of ddH_2_O, in a total volume of 20 μL. The PCR conditions for amplification were 95 °C for 5 min, 35 cycles of 95 °C for 30 s, 55 °C for 60 s or 90 s, and extension at 72 °C for 1 min, followed by 72 °C for 10 min for a final extension. To investigate whether p17-modulated expression of TKs5 and Rab40b is regulated transcriptionally, total RNA was isolated from p17-transfected cells using TRIzol kit. Quantitative real time RT-PCR with iQTM SYBR^®^ Green Supermix kit (Bio-Rad, Hercules, FL, USA) was described previously [16]. The glyceraldehyde-3-phosphate dehydrogenase (GAPDH) gene was used as an internal control.

In order to understand how p17 regulate the formation of invadopodia in B16-F10 cancer cells, pcDNA3.1-PTEN_C124A_ (a dominant-negative), pcDNA3.1-Rab40b, and pcDNA3.1-TKs5 were constructed. If this position is mutated, PTEN will lose its function of removing phosphate groups. The pCI-neo-p17 and pcDNA3.1-p17 plasmids have been described previously [17,26]. PCR products were purified and subcloned into the respective sites of the pcDNA3.1 vector.

### 2.4. shRNAs

The shRNAs were obtained from the RNAi core facility (Academia Sinica, Taipei, Taiwan). shRNA sequences are shown in Supplementary Table S3. In this work, B16-F10 cancer cells were seeded into 6 cm cell culture dishes. At about 75% confluence, cells were transfected with respective plasmids or shRNAs using Lipofectamine reagent according to the manufacturer’s protocol (Invitrogen, Carlsbad, CA, USA).

### 2.5. Transient Transfection

In this work, cells were transfected or cotransfected with the respective shRNAs or plasmids for 24 h. The scramble plasmid was used as a negative control. Cell viability measurements with the MTT assay were conducted following transfections with the respective shRNAs or plasmids. The transfection efficiency was confirmed either by Western blot or immunofluorescence staining to ensure that the transfection efficiency reached 80–90%. The inhibitory effects of Tpr, p53, PTEN, Rak, and ROCK shRNAs were tested in cells. After transfecting different shRNAs into cells, samples were collected at 0, 6, 12, 18, and 24 h, respectively. The inhibitory effect was confirmed by Western blotting. Additionally, the GFP of pGFP-V-RS vector was expressed, and the transfection efficiency can be confirmed by fluorescence.

### 2.6. Wound Healing Assays, Invadopodia Detection and Gelatin Degradation Assay

To investigate whether p17 inhibits the migration of B16-F10 cancer cells, the wound healing assay was performed using the pipette tip scratching method. Then, 24 h after transfection with pcDNA3.1-p17, scratch lines were recorded and cells were photographed and recorded. B16-F10 cells were cotransfected with the pcDNA3.1-p17 or shRNAs (Tpr, p53, PTEN, Rak, or ROCK) for 24 h. Since p53, Rak, and ROCK have been suggested to be positive regulators of PTEN, knockdown of these molecules with shRNAs was carried out [26]. The scratch test was used to test and record the migration distances of B16-F10 at 12 and 24 h post transfection. Scratch lines were recorded and cells were photographed and recorded at 0, 12, and 24 h, respectively.

To explore how p17 protein modulates signaling pathways to affect the formation of invadopodia, colocalization of cortactin and β-actin by immunofluorescence staining and gelatin degradation assays were carried out as described previously [29]. B16-F10 cells were cultured on 18 by 18 mm coverslips, followed by transfection with the respective plasmids. Cells were washed twice with 1× PBS and then fixed at the indicated times with 4% paraformaldehyde (Alfa Aesar, Haverhill, MA, USA) for 20 min at room temperature. Next, fixed cells were incubated in PBS with 0.1% Triton X-100 for 10 min. The cells were washed twice with 1× PBS and blocked with Superblock T20 solution (Thermo Scientific, Bellefonte, PA, USA) at room temperature for 1 h. After two washes with 1× PBS, cells were incubated with the cortactin primary antibodies at 4 °C overnight. The solution was removed, and cells were washed three times in PBS, 5 min for each wash. Next, the cells were incubated with β-actin primary antibody (DyLight 554 Phalloidin) and the secondary antibodies overnight at 4 °C overnight (in the dark). Cell nuclei were stained with 49,6-diamidino-2-phenylindole (DAPI) for 10 min. The cells were washed three times with 1× PBS and observed under the confocal microscope (Olympus FV1000, Tokyo, Japan). The gelatin degradation assay makes it possible to assess and quantify cellular protrusions and is crucial in the study of cell invasion. In the process of this assay, fluorophore-conjugated gelatin coated coverslips were prepared and the gelatin coating was carried out as homogeneously as possible. Briefly, the coverslips were washed with 1N hydrochloric acid for 12–16 h, washed with water and sterilized with 70% ethanol. The coverslips were then incubated with 50 μg/mL poly-L-lysine (Merck Ltd. Taipei, Taiwan) for 20 min at room temperature. The coverslips were washed twice with PBS and fixed with ice-cold 0.5% glutaraldehyde (Alfa Aesar) for 15 min, followed by washing with PBS. The coverslips were placed upside down on 80 μL of fluorescent gelatin matrix (0.2% gelatin and Alexa-Fluor-gelatin; 8:1) and incubated at room temperature for 15 min. The coverslips were washed twice with PBS and incubated with the remaining reaction matrix with PBS containing 5 mg/mL sodium borohydride for 10 min followed by two rounds of washing with PBS. B16-F10 cells were then seeded in coverslips containing gelatin matrix until cell confluence reached about 70%. Cells were cotransfected with p17 and the respective plasmids and shRNAs for 24 h. The cells on the coverslip were washed twice with PBS and fixed with 4% formaldehyde at room temperature for 20 min. Next, the fixed cells were washed twice with PBS and blocked in commercially available blocking buffer with final concentration (0.1–0.3%) of Triton X-100 at room temperature for 10–20 min. The cells were washed twice with 1× PBS to remove the remaining Triton X-100 and were immunostained with β-actin primary antibody (DyLight 554 Phalloidin) overnight. Finally, cells were observed under a confocal microscope (Olympus FV1000, Tokyo, Japan) for β-actin, to determine whether the fluorescent matrix was decomposed by invadopodia (no fluorescence produced) and to record and measure regions where the cells degraded the matrix, leaving behind areas that lacked fluorescence.

### 2.7. Coimmunoprecipitation (Co-IP) Assay

Coimmunoprecipitation assays were performed using a Catch and Release Reversible Immunoprecipitation System (Millipore, Merck Ltd., Taipei, Taiwan), as described previously [17]. Briefly, 6-well plates were seeded with 5 × 10^5^ B16-F10V cancer cells. The cells were cultured in DMEM containing 10% FBS overnight. Cells were transfected with the respective plasmids. Cell lysates were collected 24 h post transfection and washed twice with 1× PBS and scraped in 200 μL of CHAPS lysis buffer (40 mM HEPES (pH 7.5), 120 mM NaCl, 1 mM EDTA, 10 mM pyrophosphate, 10 mM glycerophosphate, 50 mM NaF, and 0.3% CHAPS). Then, 1000μg of cellular proteins were incubated with 4 ug of the respective antibodies at 4 °C overnight. The immunoprecipitated proteins were analyzed by SDS-PAGE and Western blot assay with the respective antibodies.

### 2.8. Cell Lysate Preparation and Western Blot Analysis

The B16-F10 cancer cell line was cultured in 6-well culture plates one day before infection with ARV or transfection with the respective constructs, as described above. Cells were collected and washed twice with 1× PBS with lysis buffer (Cell signaling). The concentration of proteins in cell lysates was determined by Bio-Rad Protein assay (Bio-Rad, USA). The sample was mixed with 2.5× sample buffer dye, boiled in the water bath for 15 min, and electrophoresed in 10–15% sodium dodecyl sulphate (SDS)-polyacrylamide gel. Western blot assay was performed with the respective primary antibody and horseradish peroxidase secondary antibody conjugate to analyze expression levels of each individual protein. After membrane incubation with enhanced chemiluminescence (ECL plus) regent (Amersham Biosciences, Little Chalfont, UK, the Western blot bands were detected on X-ray film (Kodak, Rochester, NY, USA).

### 2.9. Statistical Analysis

Data obtained from three independent experiments are expressed as mean ± standard errors (SE). The results were analyzed for statistical significance using Duncan’s multiple range test (MDRT) using Prism 8 software (GraphPad, San Diego, CA, USA). Similar letters (a, b, c) denote no significance.

## 3. Results

### 3.1. The ARV p17 Protein Downregulates Nucleoporin Tpr and Activates the p53/PTEN Pathway in B16-F10 Cancer Cells

**Figure 1:**
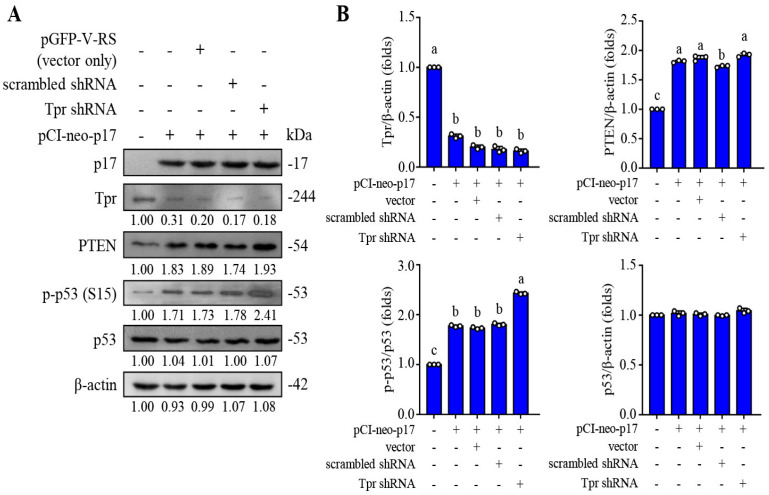
The ARV p17 protein upregulates the p53/PTEN pathway in B16-F10 cancer cells. (**A**) The expression levels of Tpr, PTEN, and p-p53 in p17-transfected cells were analyzed by Western blot. Cells were transfected with shRNAs for 6 h followed by transfection with the pCI-neo-p17 plasmid for 24 h. β-actin was included as a loading control. The image shown is from a single experiment that is representative of at least three separate experiments. Immunoblots were quantitated by densitometric analysis using ImageJ software version 1.53e and normalized to β-actin. Numbers below each lane are relative fold of the control level of a specific protein in mock-treated cells. (**B**) Densitometry analysis results for Western blotting are shown in panel A. Each value represents mean ± SE from three independent experiments, determined using Duncan’s multiple range test. Similar letters (a, b, c) denote no significance at *p* < 0.05.

Our previous study confirmed that p17 inhibits nucleoporin Tpr, thereby promoting the accumulation of p53 in the nucleus and further activating p53, PTEN, and p21 [26]. In order to confirm whether p17 has similar functions in murine melanoma cancer cells, Western blotting was used to analyze the signal changes of p17-transfected or Tpr shRNA cotransfected cells. Our results show that p17 reduces the level of nucleoporin Tpr, and the PTEN expression level and phosphorylated form of p53 are significantly increased (Figure 1A,B). If Tpr shRNA is cotransfected with pCI-neo-p17, the increased levels of PTEN p-p53(S15) are even greater (Figure 1A,B).

### 3.2. p17 Inhibits Cell Migration of B16-F10 Cancer Cells

**Figure 2:**
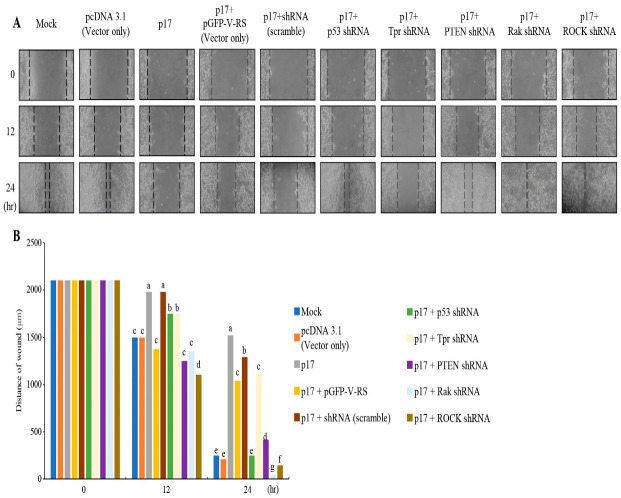
p17 inhibits cell migration of B16-F10 cancer cells. (**A**) To explore whether p17 inhibits cell migration of B16-F10 cancer cells, wound healing assays were used. Cells were transfected with the pcDNA3.1-p17 or cotransfected with shRNAs (p53, Tpr, PTEN, Rak, or ROCK-1) for 24 h. Scratch lines were recorded at 0, 12, and 24 h, respectively. The inhibitory effects of Tpr, p53, PTEN, Rak, and ROCK shRNAs were tested in cells, as shown in Supplementary Figure S1. (**B**)The scratch test was used to analyze the migration distances of B16-F10 at 0, 12, and 24 h post transfection. Scratch lines were recorded at 0, 12, and 24 h, respectively. Each value represents mean ± SE from three independent experiments, determined using Duncan’s multiple range test. Similar letters denote no significance at *p* < 0.05.

To explore whether p17 can inhibit the cell migration of B16-F10 cancer cells, wound healing assays were used. The scratch test was used to test and record the migration distances of B16-F10 at 12 and 24 h post transfection. Our results show, that compared with the control group, p17 can inhibit the migration of B16-F10 cells. Since we demonstrated previously that p17 positively regulates p53 and Rak and drives β-arrestin-mediated PTEN translocation from the cytoplasm to the plasma membrane via a ROCK-1 rependent manner [26], depletion of p53, PTEN, Rak, and ROCk-1 by the use of shRNAs was carried out. Our findings reveal that depletion of p53, PTEN, Rak, and ROCK-1 reversed p17-modulated inhibition of cell migration (Figure 2), suggesting that p17 modulated suppression of cell migration via the p53/PTEN-dependent pathway. Conversely, depletion of Tpr did not affect the p17-modulated inhibition of cell migration (Figure 2).

### 3.3. p17 Inhibits the FAK/Src Pathway and Reduces the MMP9 Level in a PTEN-Dependent Manner

**Figure 3:**
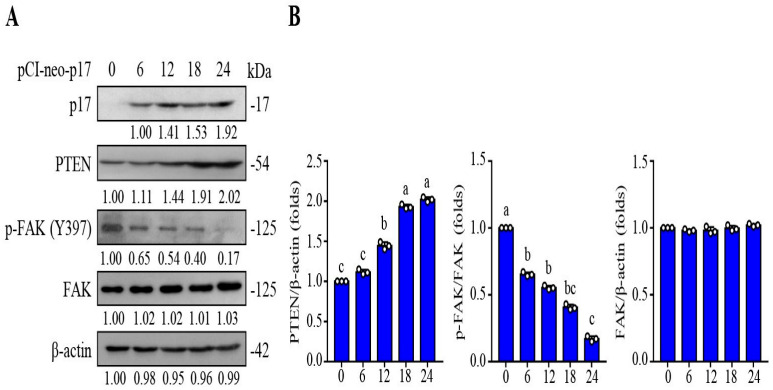
ARV p17 increases the levels of PTEN and reduces the phosphorylated form of FAK in B16-F10 cancer cells in a time-dependent manner. (**A**) The expression levels of PTEN and p-FAK in pCI-neo-p17-transfected cancer cells were analyzed. Whole cell lysates were collected at 0, 6, 12, 18, and 24 h post transfection for Western blot assays. β-actin was included as a loading control. The fold activation and inactivation indicated below each lane were normalized against the 0 h sample. The levels of indicated proteins at 0 h were considered to be 1-fold. Immunoblots were quantitated by densitometric analysis using ImageJ software and normalized to β-actin. (**B**) Densitometry analysis results for Western blots are shown in panel A. Each value represents mean ± SE from three independent experiments, determined using Duncan’s multiple range test. Similar letters (a, b, c) denote no significance at *p* < 0.05.

**Figure 4:**
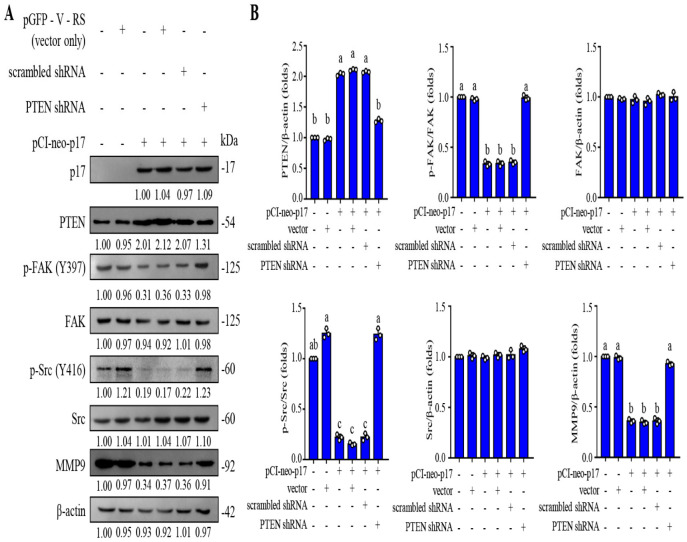
ARV p17 protein upregulates PTEN to inhibit the FAK/Src/MMP9 pathway in B16-F10 cancer cells. (**A**) The expression levels of PTEN, p-FAK, FAK, p-Src, Src, and MMP9 in pCI-neo-p17-transfected cells were analyzed by Western blots. Whole cell lysates were collected at 24 h post transfection. β-actin was used as a loading control. The fold activation and inactivation indicated below each lane were normalized against the mock control. The levels of the indicated proteins in mock treated cells were considered to be 1-fold. (**B**) Immunoblots from panel A were quantitated by densitometric analysis using ImageJ software and normalized to β-actin. Each value represents mean ± SE from three independent experiments, determined using Duncan’s multiple range test. Similar letters (a, b, c) denote no significance at *p* < 0.05.

As shown in Figure 3, an increase in the PTEN level and a decreased level of p-FAK (Y397) in B16-F10 cancer cells were observed in pCI-neo-p17 transfected cells (Figure 3A,B). We next wanted to examine whether dephosphorylation of FAK by p17 occurs through a PTEN-dependent manner. Previous reports suggested that FAK is cis- or trans-phosphorylated at Tyr-397, which provides a critical binding site for Src family kinases [30], the p85 regulatory subunit of phosphatidylinositol 3-kinase [31], and phospholipase Cγ [32]. Especially, Src binding to Tyr-397 is required for phosphorylation of Y576/Y577, which is important for full FAK activation. Subsequently, an activated FAK/Src complex mediates the phosphorylation of multiple adhesion components involved in the dynamic regulation of cell motility and invadopodia formation [8,33,34]. In this study, we found that p17 reduces levels of p-FAK(Y379), p-Src (Y416), and MMP9 (Figure 4A,B) and can be reversed in PTEN knockdown cells (Figure 4A,B), suggesting that p17 inhibits the FAK/Src pathway and reduces the MMP9 level in a PTEN-dependent manner.

### 3.4. p17 Inhibits the Formation of the FAK/Src Complex

**Figure 5:**
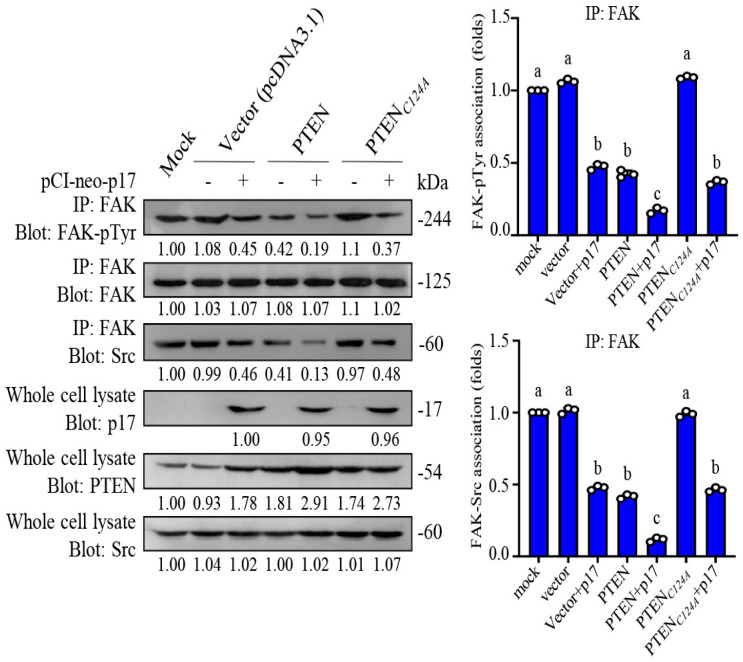
The ARV p17 activates PTEN, leading to inhibition of the FAK/Src complex formation in B16-F10 cancer cells. Coimmunoprecipitation experiments of FAK, FAK-pTyr, and Src were performed. The levels of indicated proteins in cell alone or mock transfection were considered to be 1-fold. Immunoblots from the left panel- were quantitated by densitometric analysis using ImageJ software. Each value represents mean ± SE from three independent experiments, determined using Duncan’s multiple range test. Similar letters (a, b, c) denote no significance at *p* < 0.05.

A previous report indicated that the major autophosphorylation site of FAK (Y397) is responsible for the initial in vivo association of PTEN with FAK, a prerequisite for FAK dephosphorylation by PTEN [35]. PTEN interacts with FAK and reduces its tyrosine phosphorylation at Tyr 397 [36], thereby impeding Src binding to FAK. Thus, we next investigated the molecular interaction of FAK and Src in B16-F10 cancer cells. As shown in Figure 4A, p17 significantly reduces the phosphorylation of FAK and Src, but it has not yet been confirmed whether the formation of the FAK/Src complex is inhibited. Therefore, a coimmunoprecipitation assay was used to analyze the effect of coexpression of PTEN and PTEN_C124A_ mutant in p17-transfected cells. Our coimmunoprecipitation results confirmed that p17 reduces the formation of the FAK/Src complex (Figure 5, left panel). The results show that, compared with the mock group, overexpression of p17 significantly reduced FAK tyrosine phosphorylation and reduced the amount of FAK/Src complex, while co-overexpression of PTEN can make the inhibitory effect more significant (Figure 5, left and right panels). This inhibitory effect was not altered in cells coexpressing PTEN_C124A_ mutant and p17 (Figure 5, left and right panels). Taking all findings together, p17 activates the p53/PTEN pathway to reduce levels of p-FAK(Y379), thereby reducing Src binding to Tyr-397 and the amount of the FAK/Src complex.

### 3.5. p17 Reduces the Expression Levels of TKs5, Rab40b, NCK1, and MMP9 and Suppresses the Formation of the TKs5/NCK1 Complex

**Figure 6:**
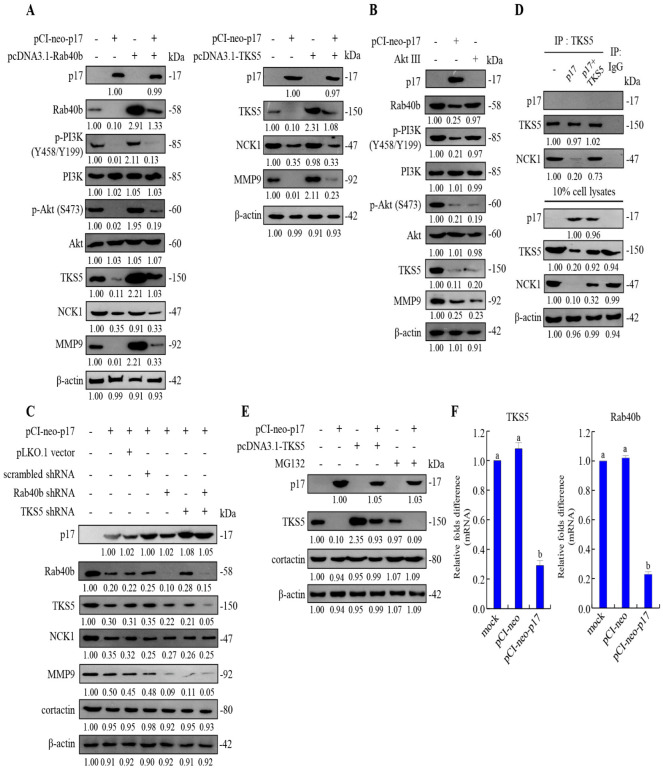
p17 reduces the expression levels of TKs5, Rab40b, NCK1, and MMP9 and suppresses the formation of the TKs5/NCK1 complex in B16-F10 cancer cells. (**A**) The levels of p-PI3K, p-Akt, TKs5, Rab40b, NCK1, and the downstream MMP9 were analyzed by Western blot assays in p17-transfected or p17/Rab40b and p17/TKs5 cotransfected cells, respectively. The level of indicated proteins in cell alone was considered to be 1-fold. The fold activation and inactivation indicated below each lane were normalized against the cell alone group. Immunoblots were quantitated by densitometric analysis using ImageJ software and normalized to β-actin. (**B**) The levels of TKs5 and MMP9 were analyzed in p17-transfected and Akt III-treated cells. (**C**) The levels of Rab40b, TKs5, NCK1, and MMP9 were analyzed by Western blot assays in p17-transfected or p17/Rab40b shRNA and p17/TKs5 shRNA cotransfected cells, respectively. (**D**) To examine whether p17 modulates the formation of the TKs5/NCK1 complex, coimmunoprecipitation assay was performed in p17-transfected or p17/Tks5-cotransfected cells. (**E**) To examine whether p17-modulated decreased levels of TKS5 are regulated by the ubiquitin-proteasome-mediated degradation pathway, the protease inhibitor MG132 (1 mM) was used to analyze the effect of MG132 on the TKS5 expression level. The levels of TKs5 were analyzed by Western blot. Cells were transfected with the pCI-neo-p17 plasmid DNA for 6 h, followed by treatment with MG132 (1 mM). (**F**) The mRNA levels of TKs5 and Rab40b genes in mock, vector, and p17-transfected cells were analyzed by qRT-PCR. The levels of the mock group in cell alone were considered to be 1-fold. Each value represents mean ± SE from three independent experiments, determined using Duncan’s multiple range test. Similar letters (a, b, c) denote no significance at *p* < 0.05. Immunoblots from panels (**A**–**E**) were quantitated by densitometric analysis using ImageJ software. The results are shown in Supplementary Figure S2.

Previous reports have shown that Src can phosphorylate TKs5 at Y557 to promote cell migration and invadopodia formation [4,34]. TKs4 and TKs5 are Src substrates. They have been identified as invadopodia organizers [34]. The initiation of invadopodia assembly has been related to the PI3K activity. Cells treated with PI3K inhibitors or PI3K-specific siRNA reduced ECM degradation and invadopodia formation [37,38]. Class I PI3K can phosphorylate phosphatidylinositol 4,5-bisphosphate [PIP (3.4)_2_] to create phosphatidylinositol (3,4,5)-trisphosphate (PIP3) [39] which will recruit kinases involved in invadopodia regulation, such as Akt [38,40]. A previous study suggested that PIP 3 can function as a precursor lipid for PIP (3.4)_2_, which localizes to invadopodia in Src-transformed fibroblasts [41]. As shown in Figure 6A (left and right panels), the levels of p-PI3K, p-Akt, TKs5, Rab40b, NCK1, and MMP9 all showed a downward trend compared with the control group after transfection with the pCI-neo-p17 construct. These findings reveal that p17 downregulates PI3K, Akt, TKs5, Rab40b, NCK1, and MMP9. Moreover, coexpressing the Rab40b protein in p17-transfected cells reversed the p17-modulated suppression of TKs5 and MMP9, while the decrease in the p-PI3K and p-Akt levels were slightly reversed (Figure 6A, left panel). The decreased level of TKs5 by p17 was not altered. Furthermore, by coexpressing the TKs5 protein, p17-modulated downregulation of MMP9 could be reversed, while the decreased level of NCK1 by p17 was not altered (Figure 6A, right panel). Interestingly, the decreased levels of TKs5 and MMP9 were also seen in Akt III-treated cells (Figure 6B), suggesting that the PI3K/Akt pathway may upregulate TKs5 and MMP9. These findings are consistent with the previous report suggesting that PTEN dephosphorylates PIP3 and FAK, and it can inhibit cell growth, invasion, migration, and focal adhesions [35]. To further study whether depletion of TKS5 and Rab40b affects the downstream MMP9, TKS5 and Rab40b shRNAs were used. The results showed that p17 reduces the levels of MMP9 and this inhibitory effect could be further elevated in TKS5 and Rab40b knockdown cells (Figure 6C).

A previous study suggested that PIP(3.4)_2_ may recruit the scaffold protein Tks5 by binding its PX domain, which will target it for localization with cortactin to the cell membrane [42]. After localization, it is thought that Tks5 regulates invadopodia formation by binding to key actin regulators such as Nck1, Nck2, NWASP, and Grb2, via its third SH3 domain [4,41]. To investigate whether p17 modulates the formation of the TKs5/NCK1 complex, a coimmunoprecipitation assay was carried out. Our data reveal that p17 reduces the amounts of the TKs5/NCK1 complex, and the inhibitory effect could be moderately reversed in cells coexpressing p17 and NCK1 (Figure 6D). The results indicate that p17 suppresses the formation of the TKs5/NCK1 complex. Having shown that the p17 protein downregulates TKs5, Rab40b, and NCK1, we next explored whether p17-modulated decreased levels of TKS5 and Rab40b are regulated by the ubiquitin-proteasome-mediated degradation pathway. Thus, the protease inhibitor MG132 was used to analyze the effect of MG132 on TKS5 expression levels. The results showed that in the presence of MG132, the expression levels of TKs5 were not altered in p17-transfected and MG132-treated cells compared with the p17 transfection group (Figure 6E). To further study whether p17 transcriptionally downregulates the TKs5 and Rab40b genes, the mRNA levels of these genes in p17-transfected B16-F10 cancer cells were quantified by qRT-PCR. As shown in Figure 6F, the mRNA levels of TKs5 and Rab40b genes were significantly reduced in p17-transfected cells, suggesting that p17 transcriptionally downregulates these genes.

### 3.6. p17 Inhibits the Formation of Invadopodia in B16-F10 Cancer Cells

**Figure 7:**
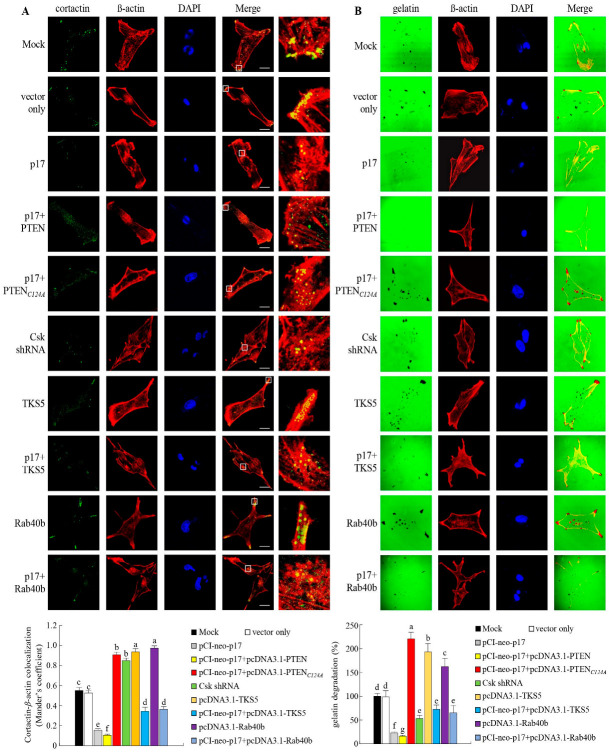
The ARV p17 protein inhibits invadopodia formation in B16-F10 cancer cells. The role of PTEN or PTEN_C124A_ mutant protein in p17-transfected cells was examined. (**A**) Cells were then fixed and processed for immunofluorescence staining of DAPI. β-actin staining (red), cortactin (green), and nuclei (blue) were observed under a confocal microscope. The invadopodia are composed of cortactin and β-actin. The yellow dots in the merged panels show invadopodia underneath the cells. The square shows the further enlargement area (100×). For quantifying the colocalization of cortactin and β-actin, at least 10 fields of 1 to 2 cells per field were taken for each sample per experiment. Each experiment was performed at least three times, while the exposure settings were unchanged during acquisition of various samples. (**B**) Images showing fluorescent gelatin (green) are from a gelatin degradation assay performed in B16-F10 cells. To quantify invadopodia activity, black areas of gelatin degradation were analyzed using ImageJ. The percent of area corresponds to degradation on a given image and normalized to β-actin. At least 10 fields of 1 to 2 cells per field were taken for each sample per experiment. Quantification of the degradation area was performed with ImageJ software. Each value represents mean ± SE from three independent experiments, determined using Duncan’s multiple range test. Similar letters (a, b, c) denote no significance at *p* < 0.05. In this study, all original images are shown in Supplementary Figure S3.

Even though it has been demonstrated that p17 modulates suppression of TKs5, NCK1, Rab40b, and MMP9 and reduces complex formation of FAK/Src and TKs5/NCK1 in B16-F10 cancer cells, the impact of p17 on the formation of invadopodia is not yet clear. Thus, we next wanted to examine whether p17 inhibits the formation of invadopodia. Colocalization of cortactin and β-actin by immunofluorescence staining and gelatin degradation assays were performed to analyze whether p17 inhibits the formation of invadopodia, as described in the Methods section. The results of colocalization of cortactin and β-actin by immunofluorescence staining reveal that the amount of invadopodia formation was significantly reduced in the pcDNA3.1-p17-transfected cells compared with the mock control group (Figure 7A). Coexpression of PTEN in p17-transfected cells significantly reduced the formation of invadopodia compared with the mock control group (Figure 7A). Overexpression of PTENC124A, TKs5, and Rab40b significantly increased the formation of invadopodia (Figure 7A), while coexpression of p17 showed a reversion to invadopodia production (Figure 7A). These findings further confirmed the impact of p17 on the formation of invadopodia. In gelatin degradation assays, we found that the fluorescent matrix was decomposed by invadopodia (no fluorescence produced) in cells overexpressing PTENC124A, TKs5, and Rab40b (Figure 7B). The formation of invadopodia could be reversed in cells coexpressing the p17 protein. p17-modulated suppression of invadopodia formation was reversed in Csk-knockdown cells. Taken together, our results reveal that the p17 protein inhibits invadopodia formation by activating the p53/PTEN pathway, suppressing the FAK/Src pathway, and downregulatingTKs5, Rab40b, NCK1, and MMP9.

## 4. Discussion

We previously demonstrated that the ARV p17 protein functions as a nucleoporin Tpr suppressor resulting in p53 accumulation in the nucleus, thereby activating the downstream molecules p21 and PTEN [26]. p17-modulated suppression of cell cycle CDK–cyclin complexes results in cell cycle retardation [17,25]. An in vivo tumorigenesis assay also showed that p17 causes a significant reduction in tumor size [17]. It was also found that p17 suppresses angiogenesis by promoting DPP4 secretion [21]. This work provides novel insights into the mechanisms underlying p17-modulated regulation of related signal pathways to suppress invadopodia formation.

Our findings reveal that p17-modulated inhibition of the formation of invadopodia in B16-F10 cancer cells relies on several different mechanisms. First, p17 activates the p53/PTEN pathway to dephosphorylate FAK at Y397 to inhibit Src binding to FAK at Y397, which prevents Src phosphorylation of TKs5 at Y557 to promote cell migration and invadopodia formation [34,37]. It was demonstrated that Tks5 functions as a tether mediating the targeting of transport vesicles containing MMP2 and MMP9 [7]. TKs5 depletion inhibited secretion of MMP2 and MMP9, suggesting that it directly mediates MMP2 and MMP9 secretion [7]. The upstream FAK signaling is involved in functions such as adhesion to the matrix and cell migration [43]. An earlier report confirmed that PTEN can compete with other signals at the FAK Y397 phosphorylation site and remove FAK phosphorylation [35], inhibiting downstream signals. In PTEN-mutated cells, regardless of whether the cells are in contact with the matrix, FAK will continue to activate Src and other pathways. If a large amount of exogenous PTEN is expressed, it can inhibit the continued activation of FAK. Clinical medicine has confirmed that in many malignant tumors, FAK mRNA transcript levels are 25% to 37% higher than those in normal cells [44]. Since FAK is involved in many cancers, in recent years, there has been a focus on developing anticancer drugs that inhibit FAK [45]. The scaffoldings protein TKs5 is essential for the assembly and function of invadopodia, and it has been confirmed that the TKs5 protein is not required for the structural composition of invadopodia, but is a key protein for stabilizing invadopodia; deletion of TKs5 in Src3T3 cells inhibits the stability of podosomes/invadopodia [6,46,47,48]. It was found that TKs5/FGD1 forms a complex, and activated CDC42 affects cell migration. If the function of these proteins is lost, MT1-MMP will be unable to function normally. Therefore, the assembly and matrix degradation of collagenolytic pseudopodia are inhibited, and the directionality and speed of tumor cell invasion through the ECM are reduced [49]. Furthermore, several studies have confirmed that reducing TKs5 protein in cancer cells in vitro or in vivo results in reduced cell invasion ability [50,51]. A decrease in Tks4 or Tks5 in cancer cells leads to reduced cell metastasis, suggesting that inhibition of pseudopodia has an additional antimetastatic effect, leading to further reduction in metastasis efficiency [52].

Second, p17 transcriptionally downregulates TKs5, Rab40b, and NCK1 to reduce the formation of TKs5/NCK1 or TKs5/Rab41b complexes, thereby suppressing the formation of invadopodia. It was found that TKs5 forms a complex with Rab40b or NCK1 to regulate invadopodia production and maturation [37]. A previous study suggested that NCK1 activity is important for invadopodia formation and degradation of the external matrix in metastatic breast cancer cells and melanoma cells [53]. TKs5 regulates invadopodia formation by binding to key actin regulators such NCK1 [4,34]. Third, in addition to the p53/PTEN and FAK/Src/Tsk5 signaling axis, our results suggest a mechanism whereby p17 exerts a negative effect on the PI3K/Akt pathway. PI3K can phosphorylate PIP (3.4)_2_ to generate phosPIP3 [39], which will recruit kinases involved in invadopodia regulation, such as Akt [38,40]. A recent study suggested that Rab40b can recognize and bind to TKs5 located at the end of invadopodia, and will carry MMPs to vesicles to guide the release of invadopodia [7]. TKs5 prompts Rab40b to intervene in the transport of MMP2/MMP9 in the formation of invadopodia [7].

Previous studies have confirmed that highly invasive cancer cells form protruding structures called invadopodia that aggregate actin and degrade ECM [54]. The structure of invadopodia is mainly composed of two different types of actin, β-actin, and cortactin, surrounded by a ring structure rich in actin-binding proteins, N-WASP, and cofilin [3,55]. The resulting invadopodia penetrates deep into the ECM and breaks it down to assist cell migration. In this work, wound healing assays, invadopodia detection by ß-actin and cortactin immunofluorescence staining, and gelatin degradation assays clearly demonstrated that p17 inhibits cell migration and the formation of invadopodia. It was suggested that after cofilin and Arp2/3 conjugates polymerize downstream proteins and actin, cortactin is dephosphorylated, which is necessary to stabilize the invadopodium precursor. In turn, stabilization is required to enable invadopodium [56]. Future studies can further explore whether p17 protein is a cofilin and Arp2/3 complex.

## Data Availability

Data are contained within the article and Supplementary Materials.

## Supplementary Materials

The following supporting information can be down loaded at: https://www.mdpi.com/article/10.3390/v16071153/s1, Figure S1: Inhibitory effects of Tpr, p53, PTEN, Rak and ROCK shRNAs were tested in cells; Figure S2: Immunoblots from Figure 6 (panels A-E) were quantitated by densitometric analysis using ImageJ software; Figure S3: All original blots and images; Supplementary Table S1: The catalog numbers and dilution factor of the respective antibodies used in this study; Supplementary Table S2: Primers used in this study; Supplementary Table S3: shRNAs used in this study.
