# Supplementary material for: The Oncolytic Avian Reovirus p17 Protein Inhibits Invadopodia Formation in Murine Melanoma Cancer Cells by Suppressing the FAK/Src Pathway and the Formation of theTKs5/NCK1 Complex
Source: Viruses. 2024 Jul 17;16(7):1153. doi: 10.3390/v16071153 (PMC11281681; doi:10.3390/v16071153)
Supplement: Supplementary file 1 [file viruses-16-01153-s001.zip › viruses-3074356-supplementary.pdf]

Supplementary Table S1. The catalog numbers and dilution factor of the respective antibodies used in this study

Antibodies	Catalog numbers	Clone name	Dilution factor	Manufacture
Mouse anti-p17*	-	-	2000	Our laboratory
Tpr	sc-100282	L-17	1000	Santa Cruz
Mouse anti-p53	2527	7F5	3000	Cell Signaling
Rabbit anti-p-p53 (S15)*	9284	-	2000	Cell Signaling
Rabbit anti-PTEN	9559	138G6	3000	Cell Signaling
Rabbit anti-p-PTEN (S380/T382/383)	9554	-	2000	Cell Signaling
Rabbit-anti-p-FAK (Y397)	8556		1500	Cell Signaling
Rabbit-anti-FAK	3285		3000	Cell Signaling
Rabbit-anti-p-Src (Y416)	6943		2000	Cell Signaling
Mouse-anti-Src	2110		3000	Cell Signaling
Mouse-anti-Csk	-		2000	BD
Rabbit-anti-TKs5	16619		3000	Cell Signaling
Rabbit-anti-Rab40b	ab82896		2000	abcam
Rabbit-anti-NCK1	2319		3000	Cell Signaling
Rabbit-antiMMP9	13667		3000	Cell Signaling
Mouse anti-cortactin	3503		3000	Cell Signaling
DyLight 554 Phalloidin	-		500	Cell Signaling
Mouse anti-β-actin	MAB1501	C4	10000	Millipore
Goat anti-mouse IgG (H+L) HRP	5220-0341	-	5000	SeraCare
Goat anti-rabbit IgG (H+L) HRP	5220-0336	-	5000	SeraCare

*Polyclonal antibodies

Supplementary Table S2. Primers used in this study

Gene	Accession number	Sequence (5′-3′)*	Expected size (bp)
Rab40b	NP_006813.1	F TAAAGCTTACGATGAGCGCCCTGGGCAG<br>R: GCGGATCCTTAAGAAATTTTGCAGCTGTTTC	837
TKs5	NM_014631 .3	F1: TAGAATTCACGATGCTCGCCTACTGCGTG (EcoR I)<br>R1: CAAAGCCGAATGCAGGGATGTCATACTC<br>F2: GAGTATGACATCCCTGCATTCGGCTTTG<br>R2: GCGTCGACCTAGTTCTTTTTCTCAAGGTAG	3318
PTEN	AB009903	F: GCGAAGCTTACCATGACAGCCATCATCAAAG (*Hind*III)	1230
		R: CGGCTCGAGTCAGACTTTTGTAATTTGTGT (*Xho*I)	
PTEN mutant C124A up		F: GCGAAGCTTACCATGACAGCCATCATCAAAG (*Hind*III)	390
		R: CCAGTTCGTCCCTTTCCACCTTTAGCGTGAA	
PTEN mutant C124A down		F: AGCAATTCACGCTAAAGCTGGAAAGGGACG	840
		R: CGGCTCGAGTCAGACTTTTGTAATTTGTGT (*Xho*I)	

Underlines in each primer indicate the restriction sites.

The restriction sites were designed in the primers for cloning.

Supplementary Table S3. shRNAs used in this study

	Cat. No.	Sequence (5′-3′)
Tpr	TG308677	GGTGAAGATAGTAATGAAGGAACTGGTAG
p53	TG320558	CTCAGACTGACATTCTCCACTTCTTGTTC
PTEN	TG320498	CTTGACCAATGGCTAAGTGAAGATGACAA
Rak	TG517143	TGGTCTCAAGAGGCAGACAAGTCAGTAGT
Rock-1	TG309775	CCAGAGTCAAGAATTGAAGGTTGGCTTTC
Csk	TRCN0000000804	CGAGGAGGTGTACTTTGAGAA
Rab40b	TRCN0000047529	CGGCATTGATCGATGGATTAA
TKS5	TRCN0000135150	CATCTATGAGAATGAGGGCTT

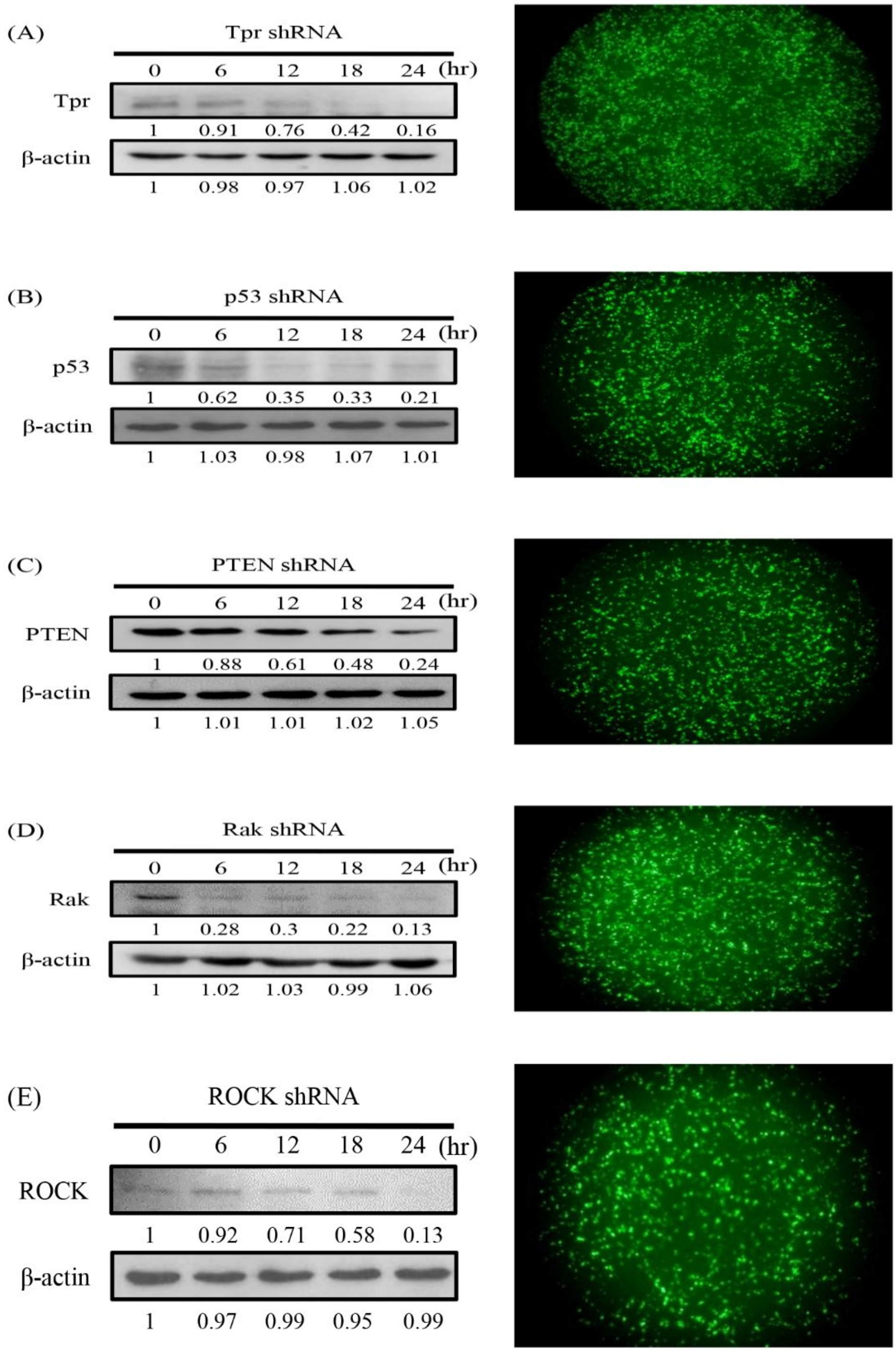

Figure S1. Inhibitory effects of Tpr, p53, PTEN, Rak and ROCK shRNAs were tested in cells. After transfecting different shRNAs into cells, samples were collected at 0, 6, 12, 18 and 24 hours respectively. The expression of shRNAs was confirmed by expressing fluorescent proteins and the inhibitory effect was confirmed by Western blotting. (A) Western blotting was used to analyze the inhibitory effect of Tpr shRNA. The results showed that Tpr expression was significantly inhibited 12 hours after transfection. (B) Western blotting was used to analyze the inhibitory effect of p53

shRNA. The results showed that the expression of p53 was significantly inhibited 6 hours after transfection. (C) Western blot analysis of the inhibitory effect of PTEN shRNA showed that protein expression was significantly inhibited 18 hours after transfection. (D) The inhibitory effect of Rak shRNA was analyzed by Western blotting method. The results showed that Rak expression was significantly inhibited 6 hours after transfection. (E) Western blot analysis of the inhibitory effect of ROCK shRNA showed that ROCK expression was significantly inhibited 12 hours after transfection.

A. Left panel

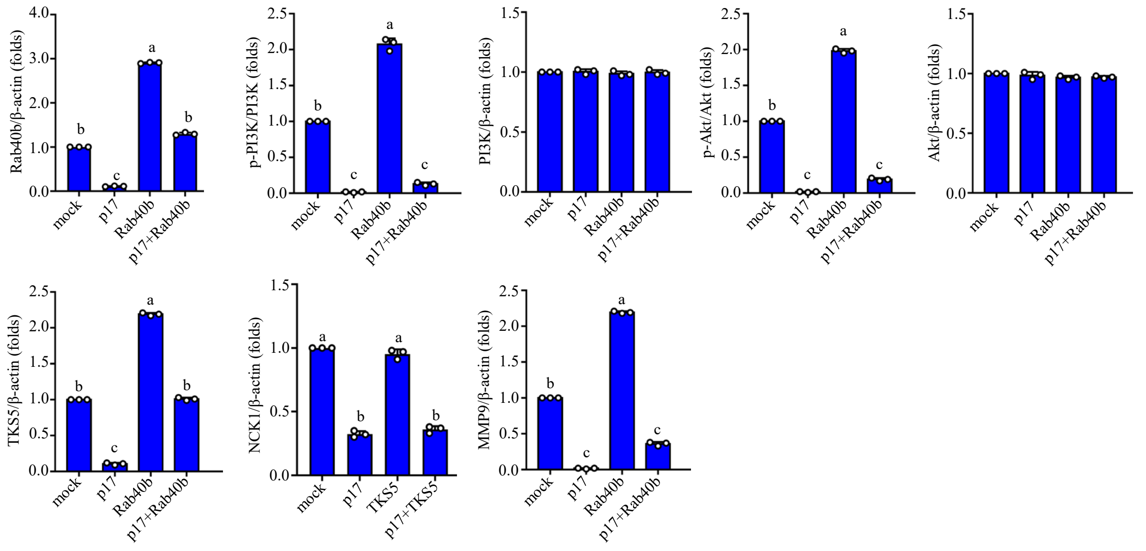

A. Right panel

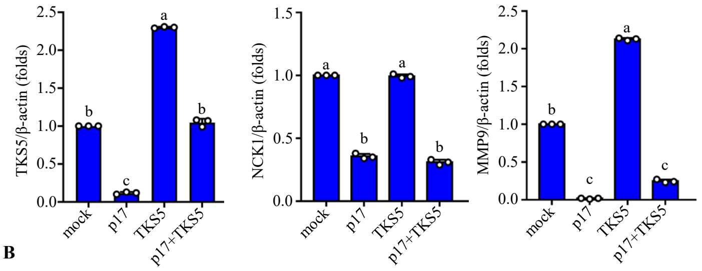

B

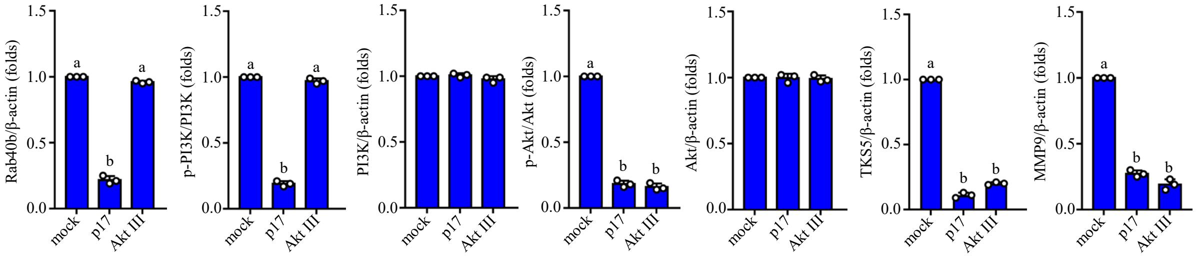

C

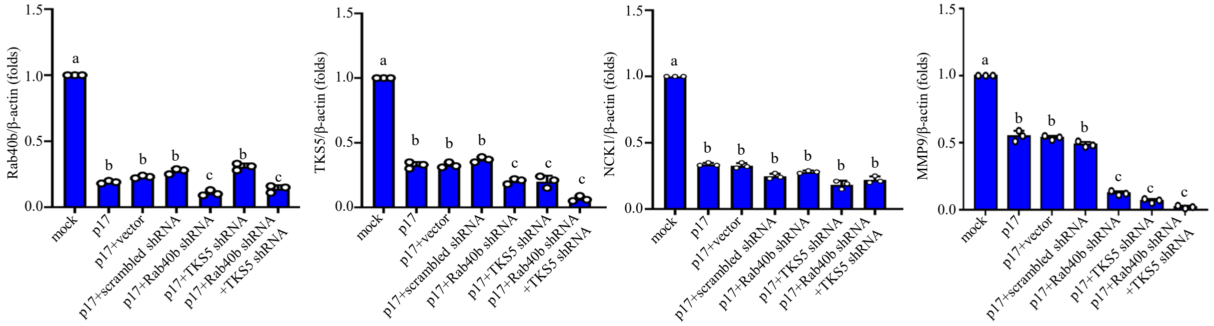

D

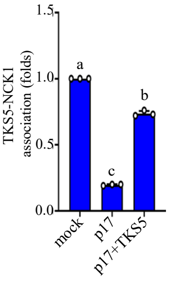

E

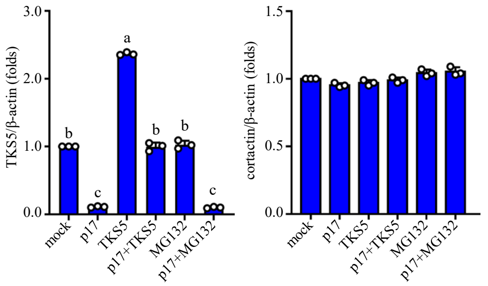

Figure S2. Immunoblots from Figure 6 (panels A-E) were quantitated by densitometric analysis using ImageJ software.

Figure 1

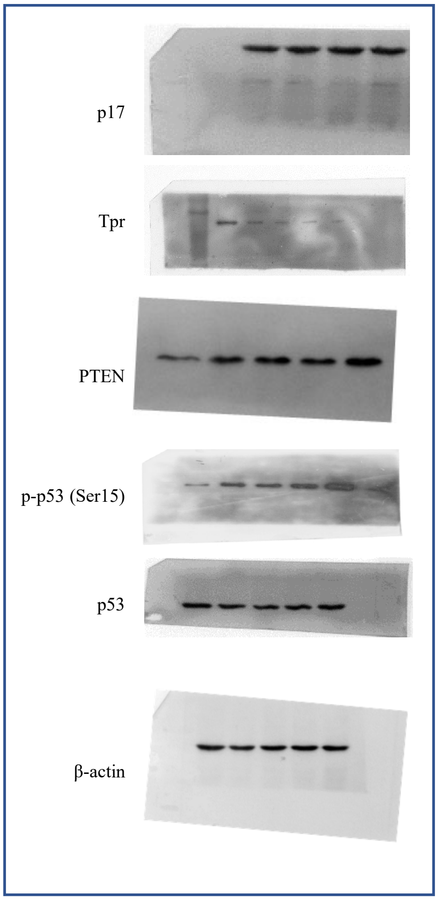

Figure 2

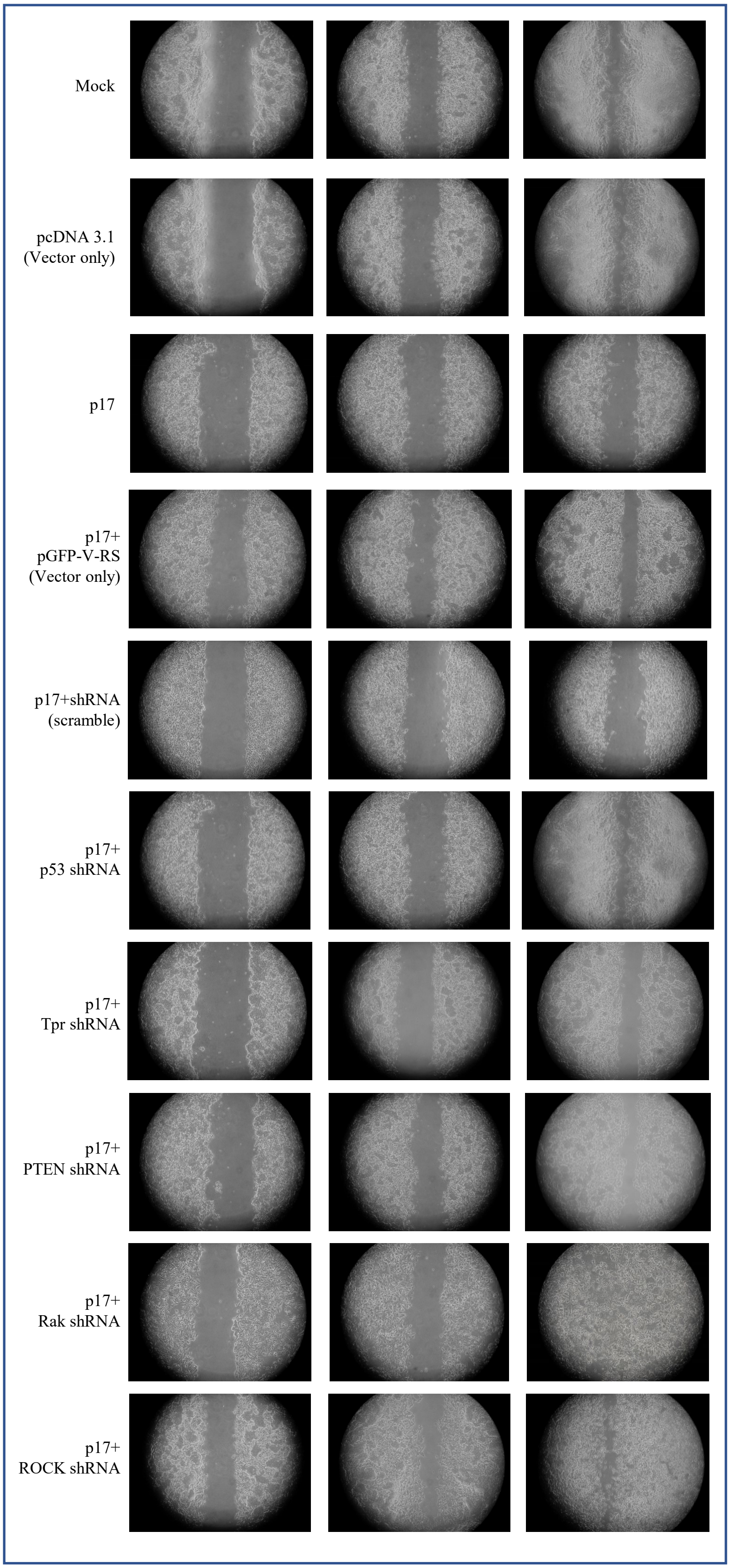

Figure 3

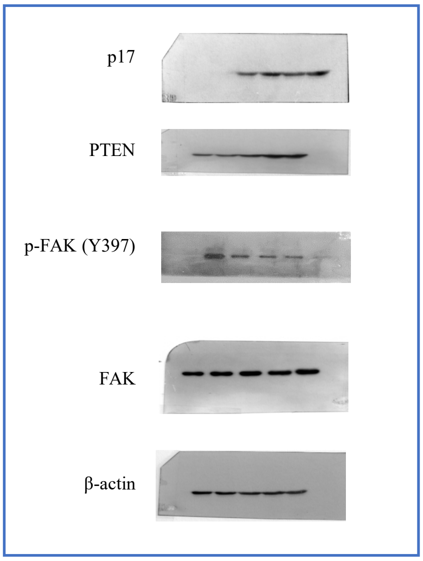

Figure 4

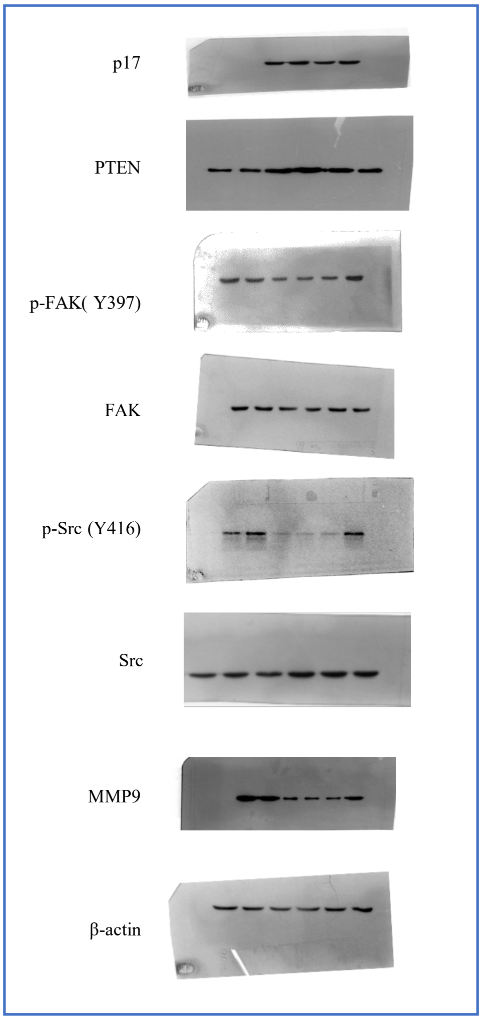

Figure 5

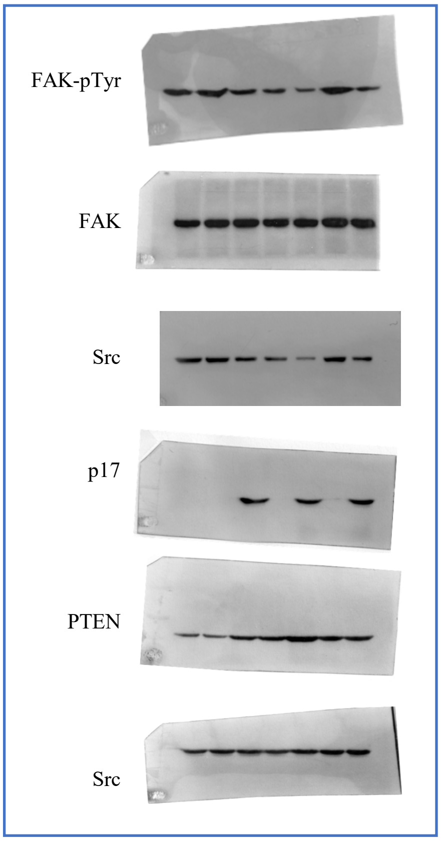

Figure 6A-1

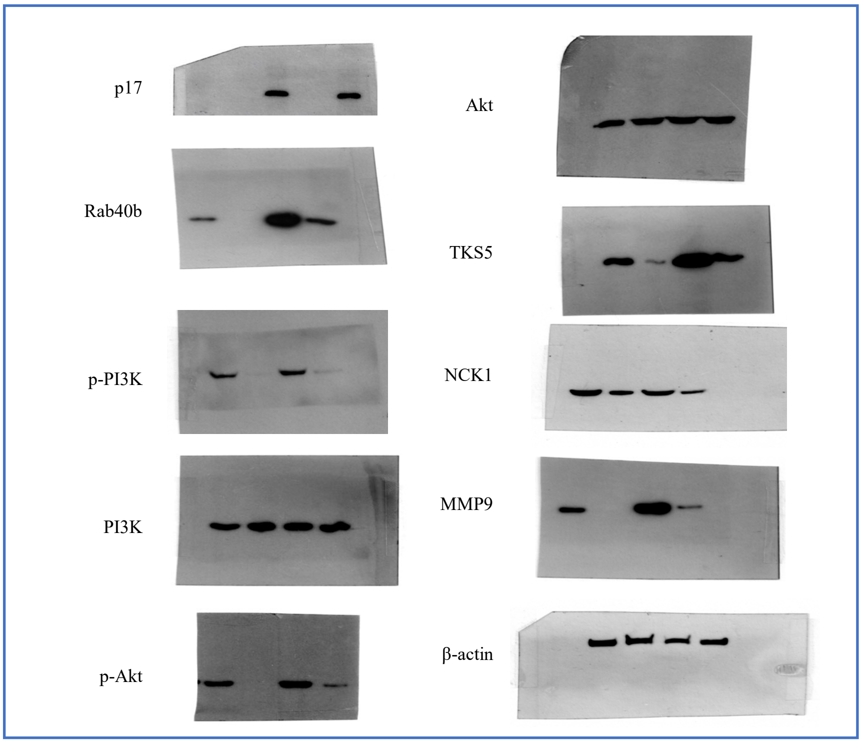

Figure 6A-2

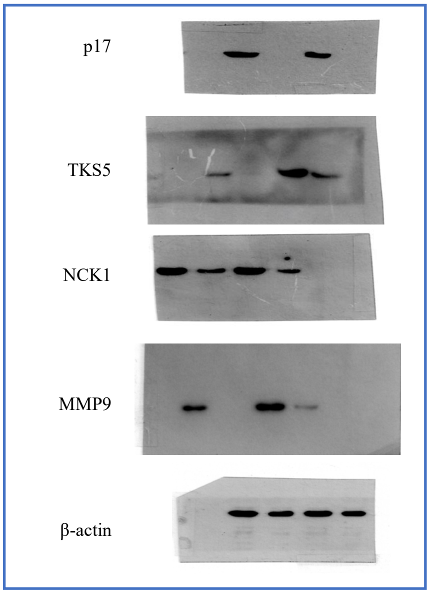

Figure 6B

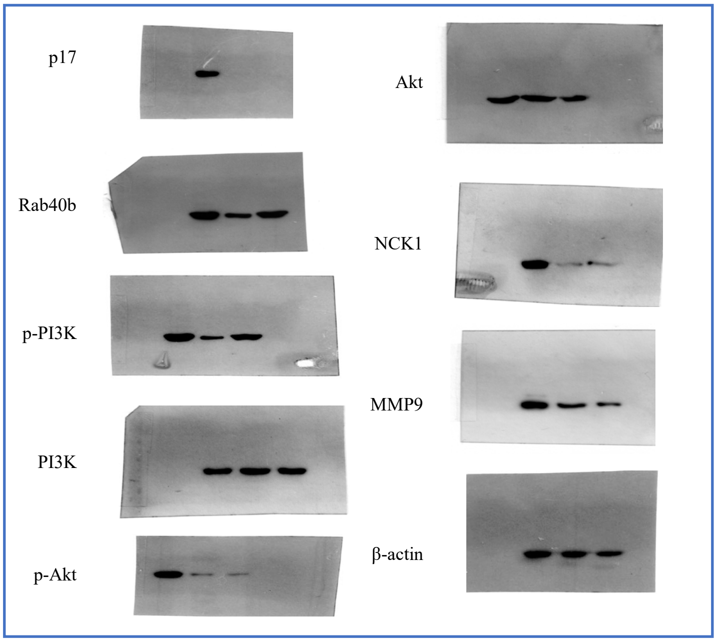

Figure 6C

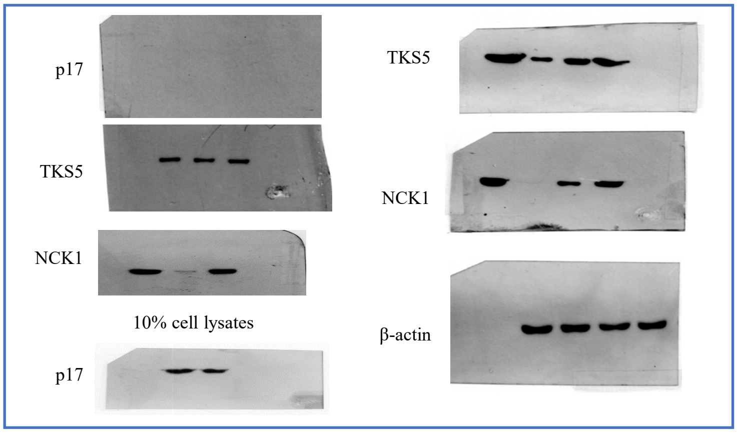

Figure 6D

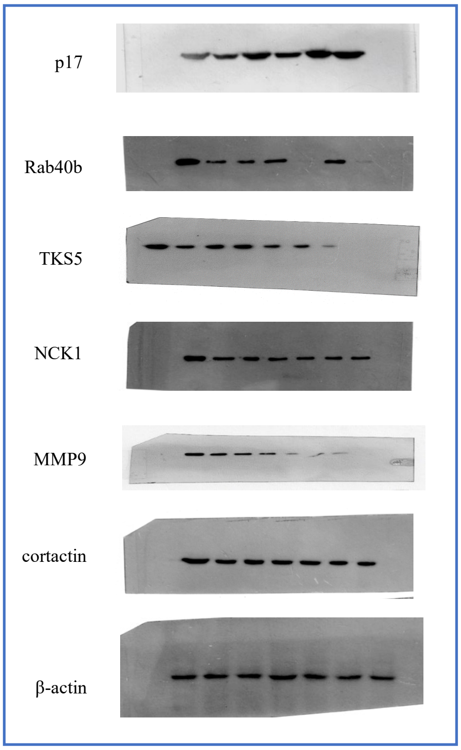

Figure 6E

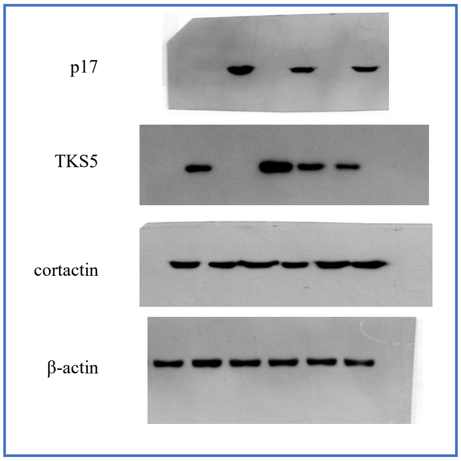

Figure 7

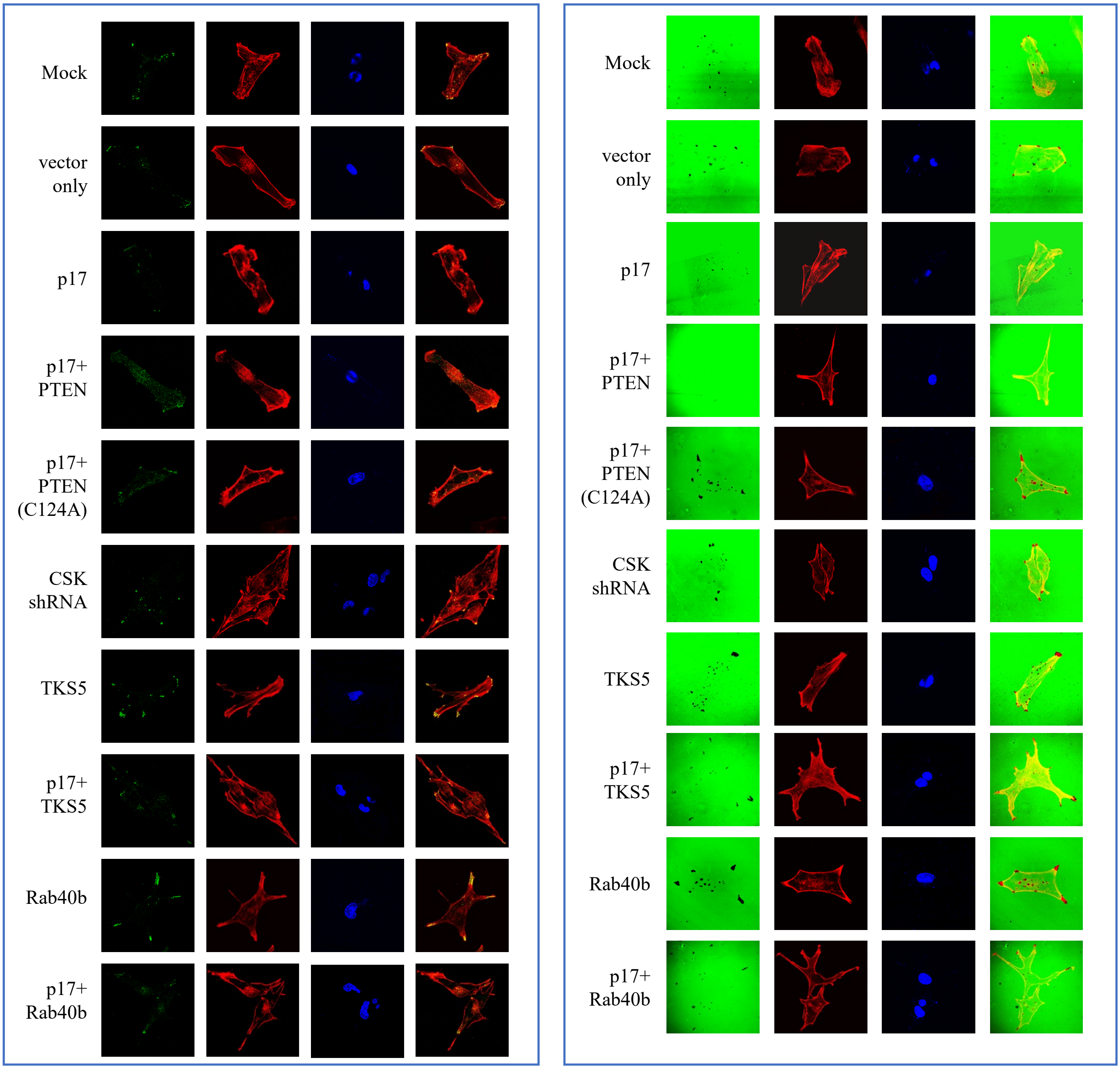

Figure S3. All original blots and images.
